# Amphiphilic Fluorinated Block Copolymer Synthesized by RAFT Polymerization for Graphene Dispersions

**DOI:** 10.3390/polym8030101

**Published:** 2016-03-22

**Authors:** Hyang Moo Lee, Suguna Perumal, In Woo Cheong

**Affiliations:** 1 School of Applied Chemical Engineering, Kyungpook National University, 80 Daehakro, Bukgu, Daegu 41566, Korea; hnctk3@naver.com (H.M.L.); suguna.perumal@gmail.com (S.P.); 2 Research Institute of Advanced Energy, Kyungpook National University, 80 Daehakro, Bukgu, Daegu 41566, Korea; 3 Department of Nano-Science and Technology, Graduate School, Kyungpook National University, 80 Daehakro, Bukgu, Daegu 41566, Korea

**Keywords:** graphene, block copolymer, RAFT polymerization, dispersion

## Abstract

Despite the superior properties of graphene, the strong π–π interactions among pristine graphenes yielding massive aggregation impede industrial applications. For non-covalent functionalization of highly-ordered pyrolytic graphite (HOPG), poly(2,2,2-trifluoroethyl methacrylate)-*block*-poly(4-vinyl pyridine) (PTFEMA-*b*-PVP) block copolymers were prepared by reversible addition-fragmentation chain transfer (RAFT) polymerization and used as polymeric dispersants in liquid phase exfoliation assisted by ultrasonication. The HOPG graphene concentrations were found to be 0.260–0.385 mg/mL in methanolic graphene dispersions stabilized with 10 wt % (relative to HOPG) PTFEMA-*b*-PVP block copolymers after one week. Raman and atomic force microscopy (AFM) analyses revealed that HOPG could not be completely exfoliated during the sonication. However, on-line turbidity results confirmed that the dispersion stability of HOPG in the presence of the block copolymer lasted for one week and that longer PTFEMA and PVP blocks led to better graphene dispersibility. Force–distance (F–d) analyses of AFM showed that PVP block is a good graphene-philic block while PTFEMA is methanol-philic.

## 1. Introduction

Graphene, a two-dimensional structured material, is a type of carbon allotrope exhibiting a hexagonal structure with *sp*^2^-bonding. This unique structure gives superior properties, such as light transparency [1], mechanical strength [2], thermal conductivity [3,4], and electron mobility [5], amongst others. Owing to these unique and superior properties, many researchers have attempted to incorporate graphene derivatives into nanocomposites [6], solar cell devices [7,8], inkjet printing [9,10], touch panels [11], flexible displays [10], and functional coatings [11]. Nevertheless, graphenes are not readily available due to their poor processability. Recently, several preparation methods for graphene have been developed, such as chemical vapor deposition (CVD) [12], mechanical exfoliation [13,14], molecular assembly [15,16], epitaxial SiC [17], and liquid-phase exfoliation [18,19]. Among these, liquid-phase exfoliation has been considered as the most efficient approach for industrial applications due to its low cost for mass production [20]. Both covalent and non-covalent functionalizations of graphene have been extensively studied in liquid-phase exfoliation. The former includes oxidation of graphite leading to defects (*i.e.*, carboxylic acid, hydroxyl, and carbonyl groups or holes) of graphite structure [20,21,22]. The latter, on the other hand, can give pristine graphenes, but it requires inclusion, dispersants, or stabilizers in order to avoid a massive aggregation of graphenes after exfoliation [23,24,25].

In order to prepare stable graphene dispersions with minimal aggregation, various types of solvents (e.g., inorganic, organic, and fluorinated oils) and surfactants (ionic, non-ionic, short, and polymeric) have been exploited [26,27,28,29]. A block copolymer dispersant for graphene dispersion has several advantages over short chain surfactants, such as better colloidal stability, minimal use of addition polymeric binders, slow migration, and controllable compatibility. To the best of our knowledge, however, there are only a few reports on block copolymer dispersants for graphene dispersion [30,31,32]. In this work, amphiphilic block copolymers of poly(2,2,2-trifluoroethyl methacrylate)-*block*-poly(4-vinyl pyridine) (PTFEMA-*b*-PVP) were synthesized by reversible addition-fragmentation chain transfer (RAFT) polymerization while molecular weights, dispersity (*Đ*_M_), and compositions of the block copolymers were finely controlled. Graphene dispersions were then prepared from highly-ordered pyrolytic graphite (HOPG) in methanol with four different types of PTFEMA-b-PVP block copolymers, and the characteristics of their dispersion states were investigated.

## 2. Materials and Methods

### 2.1. Reagents

2,2,2-Trifluoroethyl methacrylate (TFEMA, Fluorochem, Hadfield, UK) and 4-vinyl pyridine (VP, 95%, Aldrich, St. Louis, MO, USA) were used after purification by an inhibitor remover column (inhibitor removers, Aldrich). 4-Cyano-4-(phenylcarbonothioylthio)pentanoic acid (CTP, 97%, Aldrich), 1,4-dioxane (99.5%, Acros, Geel, Belgium), hexane (95%, Duksan, Ansan, Korea), tetrahydrofuran (THF, 99.5%, Duksan), toluene (anhydrous, 99.8%, Aldrich), chloroform (extra pure grade, Duksan), methanol (99.8%, Duksan), and hydrofluoric acid (50.0%, Duksan) were used as received without further purification. 2,2′-Azobisisobutyronitrile (AIBN, 98%, Junsei, Tokyo, Japan) was used after recrystallization in methanol. HOPG (graphene nanoplatelets, M-25 grade, XG Sciences, Lansing, MI, USA) was used as a source of graphene nanoplatelets. Ultra-pure water (resistivity > 18.2 MΩ·cm, Purelab, Elga, High Wycombe, UK) was used throughout the experiments.

### 2.2. Synthesis of Poly(2,2,2-trifluoroethyl methacrylate) (PTFEMA) Macro-Reversible Addition-Fragmentation Chain Transfer (RAFT) Agents

**Figure 1:**
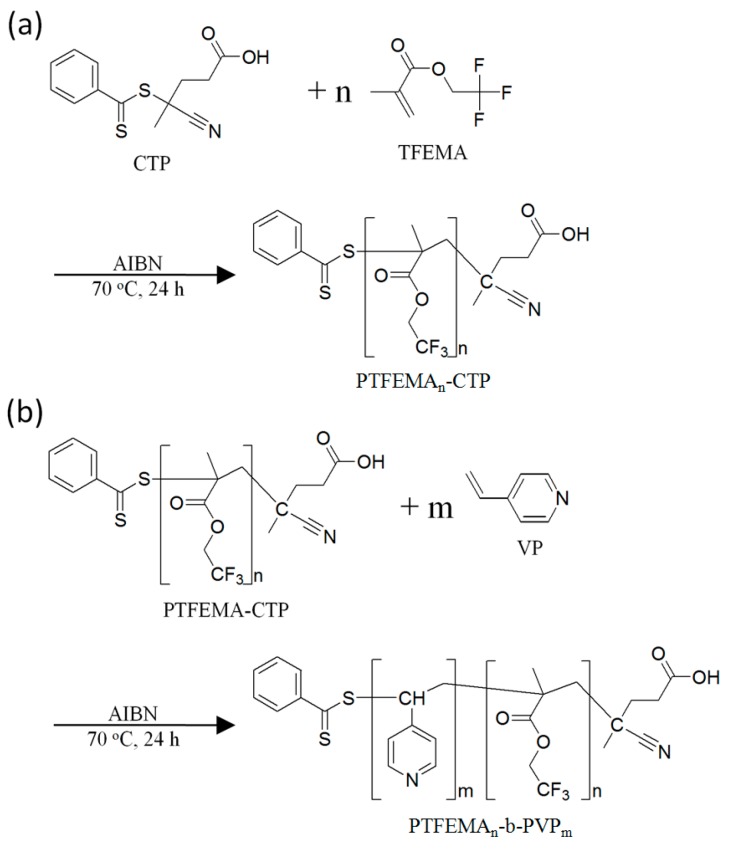
Schematic drawing of the mechanism for syntheses of (**a**) PTFEMA macro-RAFT agent (PTFEMA_n_-CTP) and (**b**) PTFEMA_n_-*b*-PVP_m_ block copolymer. CTP: 4-Cyano-4-(phenylcarbonothioylthio)pentanoic acid; TFEMA: 2,2,2-Trifluoroethyl methacrylate; AIBN: 2,2′-Azobisisobutyronitrile; VP: 4-Vinyl pyridine; PTFEMA-b-PVP: Poly(2,2,2-trifluoroethyl methacrylate)-*block*-poly(4-vinyl pyridine). RAFT: Reversible addition-fragmentation chain transfer.

RAFT polymerization was carried out to prepare the PTFEMA macro-RAFT agent. The reaction scheme is shown in Figure 1a. AIBN, CTP, and TFEMA with 1:2:100 (or 200) molar ratios were added to a 50 mL round-bottom flask along with an equal amount (g) of 1,4-dioxane as TFEMA, followed by the freeze-pump-thaw cycle three times. Following purging with N_2_ gas, the flask was sealed. The reaction mixture was then immersed in a preheated oil bath at 70 °C and stirred for 24 h. The product was purified by precipitation in hexanes three times. The resulting polymer was characterized by Fourier transform infra-red (FT-IR) spectroscopy, nuclear magnetic resonance (^1^H-NMR), and size exclusion chromatography (SEC).

### 2.3. Synthesis of Poly(2,2,2-trifluoroethyl methacrylate)-block-poly(4-vinyl pyridine) (PTFEMA-b-PVP) Block Copolymers

PTFEMA-*b*-PVP block copolymers were prepared by RAFT polymerization using PTFEMA macro-RAFT agent. The reaction scheme is shown in Figure 1b. AIBN, PTFEMA macro-RAFT agent, and VP with 1:2:100 (or 400) molar ratios were added to a 25 mL round-bottom flask with an equal amount (g) of 1,4-dioxane as the macro-RAFT agent. The freeze-pump-thaw cycle was repeated for the reaction mixture until the air bubbles disappeared during the cycle and the flask was sealed following an N_2_ gas purge. The mixture was then immersed in a preheated oil bath at 70 °C and stirred for 24 h. The resulting PTFEMA-*b*-PVP block copolymer was purified by precipitation in hexane three times. Following the synthesis procedure outlined above, four different types of block copolymers were obtained as shown in Table 1. The resulting block polymers were characterized by FT-IR and ^1^H-NMR.

### 2.4. Preparation of Graphene Dispersions

For industrial purposes, such as conductive ink or barrier coating, it is important to prepare a stable and highly concentrated graphene dispersion. To study the dispersion stability of graphene nanoplatelets, HOPG was dispersed in methanol with PTFEMA-b-PVP block copolymer as a dispersant. For the dispersion, 90.9 mg of HOPG, 9.1 mg of the block copolymer, and 20 mL of methanol were added to a vial. The concentrations of all block copolymers were fixed at 10 wt % of HOPG (0.058 wt % of methanol). The vial was then sonicated in a 40 kHz bath-type sonicator (SD 80H, S-D Ultra Sonic Cleaner, Seoul, Korea) for 2 h.

### 2.5. Adhesion Force Study Using Modified Atomic Force Microscope (AFM) Cantilever

Adhesion forces between the block (PTFEMA block or PVP block) and graphene surface were investigated by measuring Force–distance (F–d) curves obtained with an atomic force microscope (AFM, XE-7, Park System, Suwon, Korea) with modified cantilevers. To modify the cantilevers for the measurements, a cantilever (NSC-36, Park System) was etched by 2% hydrofluoric acid solution for 1 min to remove the oxide layer on the silicon surface, rinsed by ultra-pure water, dried by an air gun with gentle flowing of N_2_ gas, and then immersed into a 50 mL round-bottom flask containing a monomer solution (0.1 M, TFEMA or VP in toluene) at 110 °C. The monomer solution was kept for 2 h, and then the cantilever was rinsed with chloroform. Then, the cantilever was dried with N_2_ gas. To prepare the graphene sample, an M-25 powder sample was pelletized by using evacuable pellet dies. F–d curves were taken from 16 points on a graphene surface for one set, with 20 total sets collected. Finally, the average values of adhesion forces were calculated from the measured F–d curves.

### 2.6. Characterization

To characterize the block copolymers, molecular weights of PTFEMA macro-RAFT agents were measured by SEC (Alliance e2695, Waters, Empower Pro®, Milford, MA, USA). SEC analysis was performed with a refractive index (RI) detector and three different columns (Styragel HR3, Styragel HR4, and Styragel HR5E; Waters, Milford, MA, USA) in series. Tetrahydrofuran (THF, >99.9%, Merck, Darmstadt, Germany) was used as an eluent (35 °C, 1 mL/min). Polystyrene narrow standards (1,060 and 3,580,000 g/mol, Waters; 1,320–2,580,000 g/mol, Shodex, Tokyo, Japan) were used for the calibration. Proton nuclear magnetic resonance (^1^H-NMR) spectra of PTFEMA macro-RAFT agents and PTFEMA-*b*-PVP block copolymers were obtained using a 500 MHz NMR spectrometer (AVANCE III 500, Bruker, Karlsruhe, Germany) in chloroform-d (with 0.05% TMS, Aldrich, St. Louis, MO, USA). Fourier transform infra-red (FT-IR) spectra were obtained using an FT-IR spectrometer (IR Prestige-21, Shimadzu, Kyoto, Japan) using an ATR attachment (MIRacleA (ZnSe), Shimadzu). The polymers were dissolved in chloroform for the FT-IR measurements.

For dispersion stability, online turbidity data (Turbiscan LAB, Formulaction Co., L’Union, France) and photographic images were taken for a week. After the turbidity analyses, the supernatant graphene solution was isolated for further analyses. The graphene concentration of the supernatant solutions was measured by the gravimetric method with a microbalance (XM 1000P, Sartorius, Göttingen, Germany). For Raman spectroscopy and AFM topography, the isolated supernatant solution was spin coated on a silicon wafer under inert conditions with a spin speed of 700 rpm. In the case of pristine (without block copolymer) HOPG dispersion, there was no graphene in the supernatant solution due to the rapid sedimentation, thus the bottom precipitates were taken for analyses. Raman spectra (Almega X, Thermo scientific, Waltham, MA, USA) at 532 nm wavelength and AFM images were obtained for the precipitate sample. The AFM images were collected using an AFM (XE7, Park system) with a PPP-NCHR (Park system) cantilever in non-contact mode.

## 3. Results and Discussion

### 3.1. PTFEMA-b-PVP Block Copolymers

**Figure 2:**
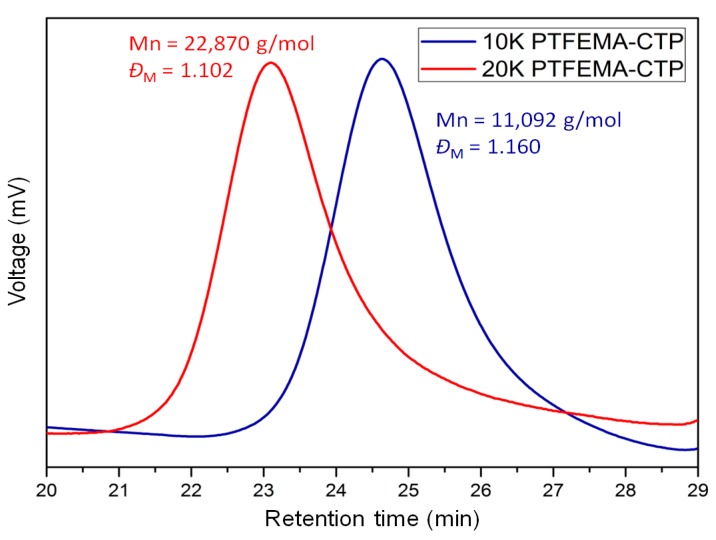
SEC elution curves for two different macro-RAFT agents: PTFEMA_66_-CTP (blue) and PTFEMA_136_-CTP (red).

In this work, four different block copolymers listed in Table 1 were prepared from stepwise RAFT polymerization. The molecular weights of two PTFEMA macro-RAFT agents were confirmed by SEC and the number average molecular weights (*M*_n_) were 11,092 g/mol (*Đ*_M_ = 1.160) and 22,870 g/mol (*Đ*_M_ = 1.102), while the degrees of polymerization were calculated as 66 and 136, respectively (refer to Figure 2). The agent with the lesser *M*_n_ was used in the second RAFT polymerization for PTFEMA-*b*-PVP block copolymers designated as **1** and **2**, and the agent with the greater *M*_n_ was used for **3** and **4**, as shown in Table 1. The molecular weights of PTFEMA-*b*-PVP block copolymers could not be measured using SEC because of the low solubility in typical SEC eluents, e.g., THF, chloroform, and *N*,*N*-dimethylformamide (DMF). Thus, the molecular weights of the block copolymers were estimated from the proton peak integration of ^1^H-NMR spectra.

**Figure 3:**
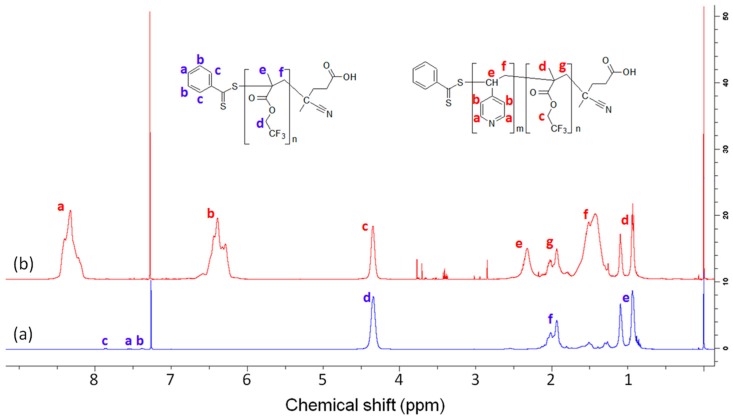
Representative ^1^H-NMR spectra for (**a**) PTFEMA_66_-CTP and (**b**) PTFEMA_66_-*b*-PVP_41_ block copolymer.

The degrees of polymerization of PVP block were obtained from the SEC results for PTFEMA macro-RAFT agents and ^1^H-NMR peak integrals for PTFEMA-*b*-PVP block copolymers. As shown in Figure 3b, peaks a and b (δ 6.39 and 8.33 ppm) are from –ArH protons in PVP block, and peak c (δ 4.34 ppm) is from –CH_2_CF_3_ protons in PTFEMA block. From the degree of polymerization for PTFEMA from SEC with the ratios of peak area a, b, and c, the length of PVP block could be calculated. The molecular weights of four PTFEMA-b-PVP block copolymers are listed in Table 1. The split peaks –CH_3_ from PTFEMA block at δ 1.10 and 0.94 ppm are also observed since the protons are hindered.

**Figure 4:**
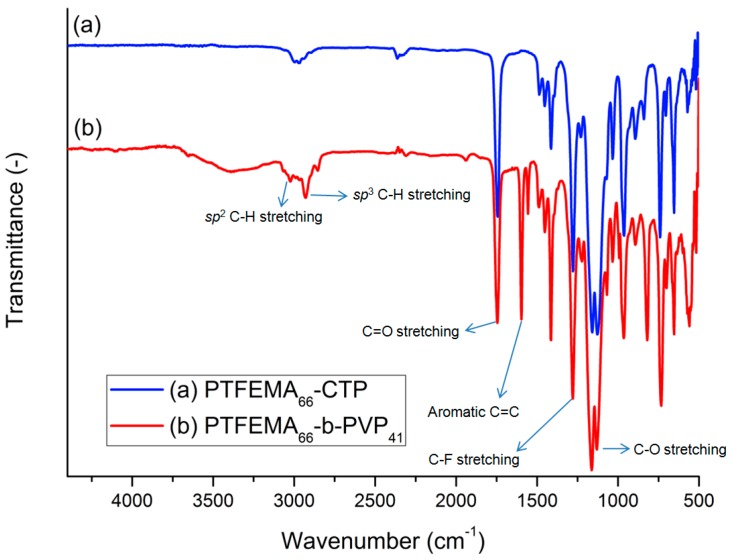
Representative FT-IR spectra for (**a**) PTFEMA_66_-CTP homopolymer and (**b**) PTFEMA_66_-*b*-PVP_41_ block copolymer.

Figure 4 shows the representative FT-IR spectra of PTFEMA_66_-CTP homopolymer and PTFEMA_66_-*b*-PVP_41_ block copolymer. As shown in the FT-IR spectra, a *sp*^3^ –C–H stretching at 3000–2940 cm^−1^, C=O stretching at 1743 cm^−1^, C–F stretching at 1278 cm^−1^, and C–O stretching for ester at 1159 and 1127 cm^−1^ were observed for the TFEMA unit. On the other hand, *sp*^2^ –C–H stretching at 3080–3000 cm^−1^ and aromatic C=C peak at 1598 cm^−1^, which correspond to the pyridine units of PVP block, are only observed in Figure 4b.

### 3.2. Adhesion Force Analyses

In order to measure the affinity of each block in PTFEMA-*b*-PVP for the surface of HOPG graphene, F–d curves were recorded using the monomer-modified AFM cantilevers. The adhesion forces were found to be 2.2 ± 0.7 nN for TFEMA and 9.3 ± 0.9 nN for VP, respectively. From the adhesion forces, one can see that VP has 4.2 times greater adhesion force than TFEMA. This suggests that PVP block is more graphene-philic than PTFEMA, despite the greater methanol solubility of PVP relative to PTFEMA. As previously determined, the lone pair electron on the nitrogen atom has an attractive interaction with pi orbitals in a graphene sheet [33]. Due to the strong attractive interaction between the VP and the graphene surface, the PVP block in the block copolymer plays the role of graphene-philic block, while PTFEMA block plays the role of lyophilic block.

### 3.3. Stability of Graphene Dispersions

**Figure 5:**
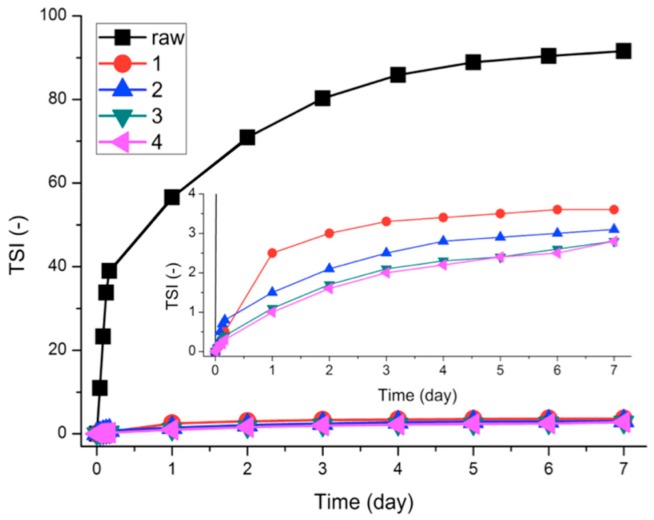
Time-evolution turbiscan stability index (TSI) curves for pristine HOPG (**raw**) and pristine HOPG with PTFEMA_66_-*b*-PVP_41_ (**1**), PTFEMA_66_-*b*-PVP_205_ (**2**), PTFEMA_136_-*b*-PVP_31_ (**3**), and PTFEMA_136_-*b*-PVP_194_ (**4**). The TSI values are calculated for the middle parts of the sample vials shown in Figure 6. The inset shows magnified TSI curves for samples **1**–**4**.

Figure 5 shows the dispersion stability of graphene dispersions in terms of turbiscan stability index (TSI), defined as follows:

(1) $$\text{TSI}=\sum_{i}\frac{\sum_{h}\left|{\text{scan}}_{i}{(\text{h})-\text{scan}}_{i-1}(\text{h})\right|}{H}$$

where H is the length of the sample, h is the height of the measure point, and scan_i_(h) is i^th^ turbiscan intensity at height h. Based on the TSI value, aggregation, precipitation, or creaming of the dispersion can be evaluated. As the dispersion becomes less stable, the TSI value increases [34]. As shown in Figure 5, the time-evolution TSI curve designated as pristine HOPG (M-25, **raw**) surges for the first few hours. Comparably, the TSI curves for the samples designated as **1**, **2**, **3**, and **4** show insignificant change with time—less than one-tenth of the variation seen in sample **raw**—indicating that the block copolymers stabilize graphene in methanol media effectively.

**Figure 6:**
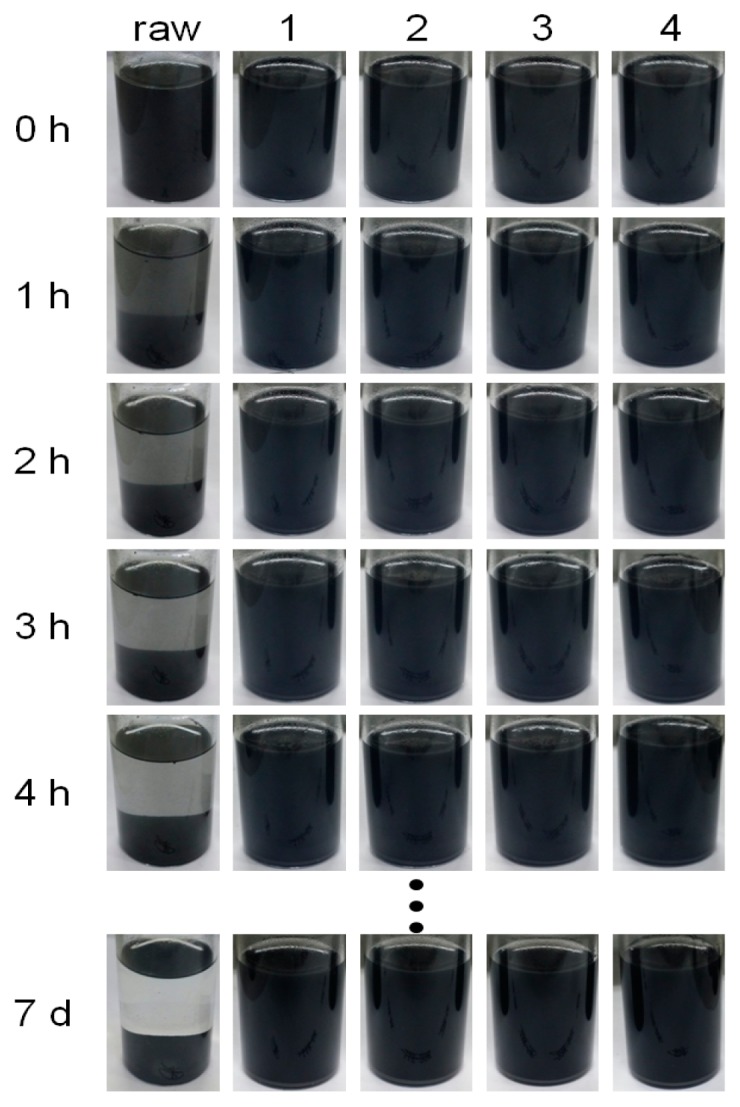
Photographic images for HOPG dispersions *versus* time obtained for pristine HOPG (M-25, **raw**), HOPG with PTFEMA_66_-*b*-PVP_41_ (**1**), PTFEMA_66_-*b*-PVP_205_ (**2**), PTFEMA_136_-*b*-PVP_31_ (**3**), and PTFEMA_136_-*b*-PVP_194_ (**4**).

Figure 6 presents photographic images of the graphene dispersions corresponding in time intervals to the TSI analyses in Figure 5. Sample **raw** settled down within 4 h. On the other hand, samples **1**, **2**, **3**, and **4** were stable even for one week. These results confirm that PTFEMA-*b*-PVP block copolymers are suitable for methanolic HOPG graphene dispersions.

Further, block copolymers **3** and **4** show little TSI value increases as compared to **1** and **2** (refer to the inset in Figure 5). As listed in Table 1, **2** has a longer PVP block length than **1**, while **3** and **4** have longer PTFEMA blocks than **1** and **2**. As demonstrated in Figure 5, in the case of the samples with the shorter PTFEMA block length (11,092 g/mol), the block copolymer with a longer PVP block length (**2**) shows better stability relative to the copolymer with a shorter PVP block length (**1**). However, for the samples with the longer PTFEMA block length (22,870 g/mol), there was no significant difference in TSI for samples **3** and **4,** despite differences in their PVP block lengths.

It has been known that the destabilization of graphene nanoplatelets in a solution is attributed to strong π–π interactions among graphene surfaces. Aggregation among the graphene nanoplatelets is thermodynamically favorable but the aggregation can be kinetically retarded by using either a stabilizer or a surfactant. PTFEMA-*b*-PVP copolymers can retard the aggregation of graphene nanoplatelets by steric hindrance among adsorbed block copolymers. PTFEMA-*b*-PVP copolymers are anchored at the basal plane of graphene by the adsorption of graphene-philic PVP blocks, while lyophilic PTFEMA blocks are stretched into methanol medium. As shown here, both PTFEMA and PVP block lengths are critical for minimizing the destabilization of graphene.

### 3.4. Graphene Nanoplatelets

The graphene concentrations of supernatant solutions were measured from the HOPG dispersions by gravimetry one week after the sonication. As shown in Table 1, the graphene concentration was in increasing order as follows: **1** < **3** < **2** < **4**. These results suggest that PVP block length is critical for maintaining stability and preventing graphene nanoplatelets from aggregation. For the preparation of a stable graphene dispersion, both longer lyophilic and graphene-philic blocks are favorable.

**Figure 7:**
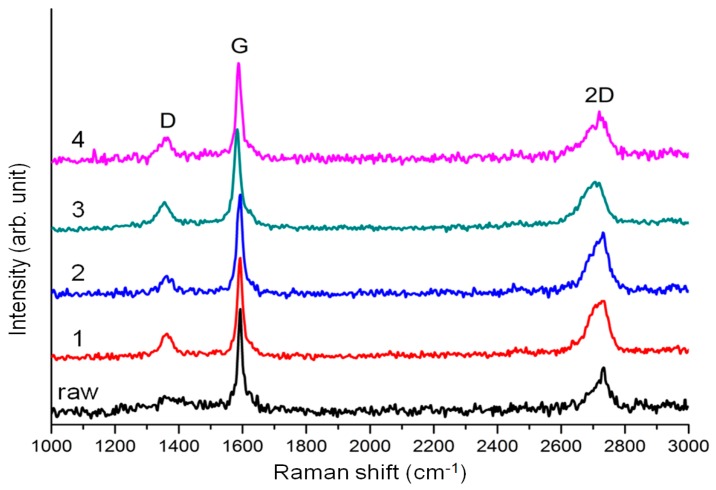
Raman spectra for supernatant solution of HOPG dispersions obtained from pristine HOPG (M-25, **raw**), HOPG with PTFEMA_66_-*b*-PVP_41_ (**1**), PTFEMA_66_-*b*-PVP_205_ (**2**), PTFEMA_136_-*b*-PVP_31_ (**3**), and PTFEMA_136_-*b*-PVP_194_ (**4**).

In order to study the graphene structure after sonication, Raman and AFM analyses were carried out. In the Raman spectra shown in Figure 7, the D bands are observed at 1360 cm^−1^. When the hexagonal structure of graphene is disturbed, the intensity of the D band increases. Defectless or perfect graphene usually has an *I*_D_/*I*_G_ value of zero, while graphene oxide has an *I*_D_/*I*_G_ value of >0.9 [35]. In addition the G band and the 2D band, peaks appear at 1590 and 2700 cm^−1^, respectively. The thickness of graphene can be estimated from the *I*_2D_/*I*_G_ value, where the *I*_2D_/*I*_G_ value for monolayer graphene is >2.0, and the *I*_2D_/*I*_G_ value for pristine graphite is <0.4 [36,37,38]. From Figure 7 and Table 2, it is seen that the graphene nanoplatelets are almost like graphite, since the *I*_2D_/*I*_G_ value for pristine graphite (HOPG, **raw**) is 0.4281. Compared to this, the *I*_2D_/*I*_G_ values for samples **1**, **2**, **3**, and **4** are slightly increased with the range from 0.4692 to 0.6125. On the other hand, the *I*_D_/*I*_G_ value of sample **raw** is 0.14735, but the *I*_D_/*I*_G_ values for **1**, **2**, **3**, and **4** are in the range from 0.17071 to 0.26248. The increases of the *I*_D_/*I*_G_ for graphene dispersions with block copolymers are due to the increased number of edges in graphene nanoplatelets. Since Raman spectra for **1**, **2**, **3**, and **4** were taken from the supernatant solution of HOPG dispersions, the graphene size would be smaller than in sample **raw**. This small size of graphene has more edge sides working as defects.

**Figure 8:**
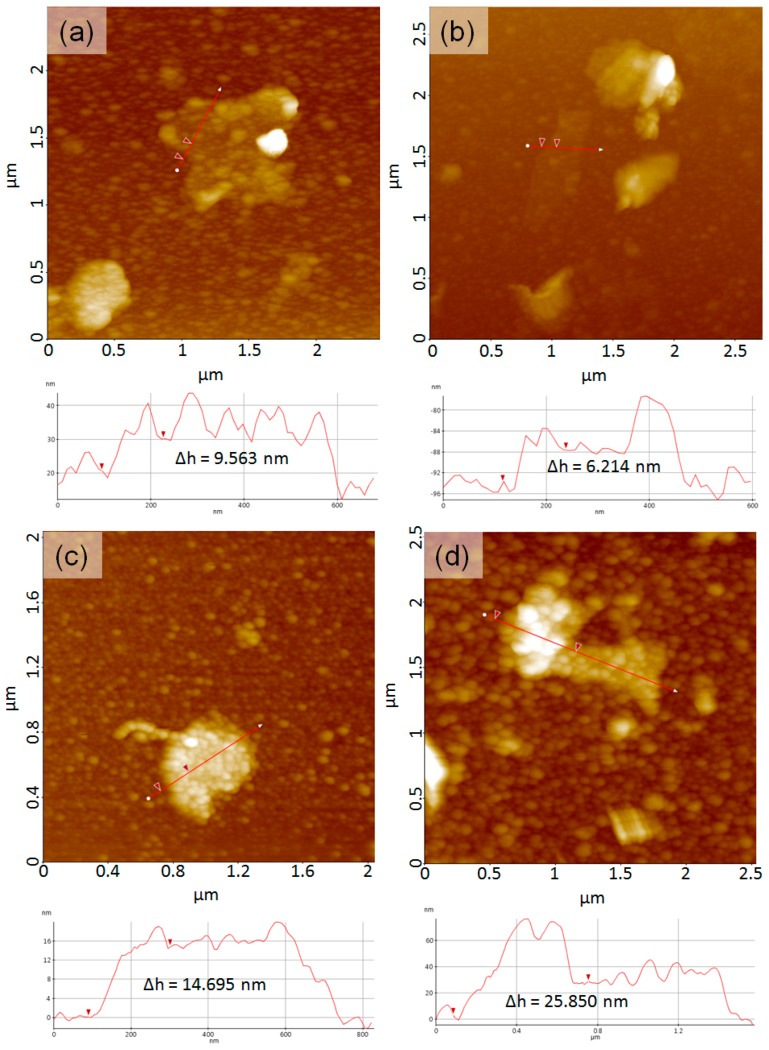
Atomic Force Microscopy (AFM) topographic images of the supernatant solution from HOPG dispersions with (**a**) PTFEMA_66_-*b*-PVP_41_, (**b**) PTFEMA_66_-*b*-PVP_205_, (**c**) PTFEMA_136_-*b*-PVP_31_, and (**d**) PTFEMA_136_-*b*-PVP_194_. Height profiles from the red line on topographic images are depicted below their corresponding images (**a**–**d**), and the Δh in height profile reveals the height diffrences between red triangles.

Figure 8 shows AFM topographic images of the supernatant solutions obtained from graphene dispersion samples. All images show multi-layered graphene nanoplatelets covered with block copolymers. The size distribution of graphene was from a few hundred nanometers to micrometers. The thickness of graphene covered with the polymer aggregates ranged about 6–25 nm, indicating that the number of graphene layers were fewer than 18–73 sheets. Both the graphene surface and the silicon background are covered with the block copolymer aggregates. As for the aggregate size of the block copolymer, sample **4** shows the largest block copolymer aggregates due to the largest molecular weight relative to the other samples.

## 4. Conclusions

Four different types of PTFEMA-*b*-PVP block copolymers were prepared by using stepwise RAFT polymerization. The molecular weights of PTFEMA-CTP homopolymers were measured by SEC, and the molecular weights of PTFEMA-*b*-PVP block copolymers were calculated by SEC and ^1^H-NMR. Pristine HOPG was dispersed in methanol by using the block copolymers, and the dispersion stability of the resulting graphene dispersions were evaluated using the Turbiscan stability index. Dispersion stability of HOPG in the presence of PTFEMA-*b*-PVP lasted one week and the graphene concentrations of the supernatant solutions ranged from 0.260 to 0.385 mg/mL. Time-dependent backscattering intensity confirmed that TFEMA-*b*-PVP copolymers could substantially retard the aggregation of graphene nanoplatelets. It can be suggested from the F–d curve results that PVP blocks would adsorb at the basal plane of graphene while PTFEMA blocks are soluble in methanol. Therefore, both PVP and PTFEMA block lengths are critical in determining both graphene concentration and dispersion stability.

## Figures and Tables

**Table 1:** The number average molecular weights and degrees of polymerization of PTFEMA-*b*-PVP block copolymers and the graphene concentrations from the supernatant solution of highly-ordered pyrolytic graphite (HOPG) dispersions with corresponding block copolymers.

Designation	*M*_n_ for PTFEMA ^1^ (g/mol)	*M*_n_ for PVP ^2^ (g/mol)	Degree of polymerization	Graphene concentration ^3^ (mg/mL)
1	11,092	4,338	PTFEMA_66_-*b*-PVP_41_	0.260
2	11,092	21,581	PTFEMA_66_-*b*-PVP_205_	0.350
3	22,870	3,309	PTFEMA_136_-*b*-PVP_31_	0.275
4	22,870	20,410	PTFEMA_136_-*b*-PVP_194_	0.385

^1^ determined by size exclusion chromatography (SEC); ^2^ determined by proton nuclear magnetic resonance (^1^H-NMR) peak integration and SEC data of PTFEMA; ^3^ from the supernatant solution of HOPG methanolic dispersion one week after sonication.

**Table 2:** *I*_2D_/*I*_G_ and *I*_D_/*I*_G_ values from Raman spectra for monolayer graphene, graphite, graphene oxide, pristine HOPG (M-25, **raw**), HOPG with PTFEMA_66_-*b*-PVP_41_ (**1**), PTFEMA_66_-*b*-PVP_205_ (**2**), PTFEMA_136_-*b*-PVP_31_ (**3**), and PTFEMA_136_-*b*-PVP_194_ (**4**).

Designation	*I*_2D_/*I*_G_	*I*_D_/*I*_G_
Monolayer graphene	>2.0	–
Graphite	<0.4	–
Graphene oxide	–	>0.9
M-25, **raw**	0.4281	0.1474
**1**	0.5601	0.2250
**2**	0.6125	0.1707
**3**	0.4692	0.2625
**4**	0.4983	0.2307
